# Small RNA sequencing for secondary metabolite analysis in *Persicaria minor*

**DOI:** 10.1016/j.gdata.2017.05.014

**Published:** 2017-05-13

**Authors:** Abdul Fatah A. Samad, Nazaruddin Nazaruddin, Muhammad Sajad, Jaeyres Jani, Abdul Munir Abdul Murad, Zamri Zainal, Ismanizan Ismail

**Affiliations:** a School of Biosciences and Biotechnology, Faculty of Science and Technology, Universiti Kebangsaan Malaysia, 43600 UKM Bangi, Selangor, Malaysia; b Institute of Systems Biology, Universiti Kebangsaan Malaysia, 43600 UKM Bangi, Selangor, Malaysia; c Department of Chemistry, Faculty of Mathematics and Natural Sciences, University of Syiah Kuala, Darussalam, Banda Aceh 23111, Indonesia; d BioEasy Sdn. Bhd. and ScienceVision Sdn. Bhd., Setia Alam, Seksyen U13, 40170 Shah Alam, Selangor, Malaysia; e Department of Plant Breeding and Genetics, University College of Agriculture & Environmental Sciences, The Islamia University of Bahawalpur, Punjab, Pakistan

**Keywords:** Small RNA sequencing, *Persicaria minor*, *Fusarium oxysporum*, Secondary metabolite

## Abstract

*Persicaria minor* (kesum) is an important medicinal plant and commonly found in southeast countries; Malaysia, Thailand, Indonesia, and Vietnam. This plant is enriched with a variety of secondary metabolites (SMs), and among these SMs, terpenoids are in high abundance. Terpenoids are comprised of many valuable biomolecules which have well-established role in agriculture and pharmaceutical industry. In *P. minor*, for the first time, we have generated small RNAs data sets, which can be used as tool in deciphering their roles in terpenoid biosynthesis pathways. Fungal pathogen, *Fusarium oxysporum* was used as elicitor to trigger SMs biosynthesis in *P. minor.* Raw reads and small RNA analysis data have already been deposited at GenBank under the accessions; SRX2645684 (*Fusarium*-treated), SRX2645685 (*Fusarium*-treated), SRX2645686 (mock-infected), and SRX2645687 (mock-infected).

**Table:** 

Specifications	
Organism/cell line/tissue	*Persicaria minor* (Leaf)
Sex	Not applicable
Sequencer or array type	HiSeq 2500™ (Rapid Run)
Data format	Raw (FASTQ)
Experimental factors	Controlled growth chamber
Experimental features	Small RNA expression and discovery
Consent	Public (No ethics approval needed for this project)
Sample source location	Selangor, Malaysia (3° 16′14.63″ N, 101° 41′ 11.32″ E)

## 1 Direct link to deposited data

https://www.ncbi.nlm.nih.gov/sra/SRX2645684 (*Fusarium-*treated)

https://www.ncbi.nlm.nih.gov/sra/SRX2645685 (*Fusarium*-treated)

https://www.ncbi.nlm.nih.gov/sra/SRX2645686 (Mock-infected)

https://www.ncbi.nlm.nih.gov/sra/SRX2645687 (Mock-infected)

## 2 Experimental design, materials and methods

### 2.1 Plant materials

*P. minor* explants were propagated and grown in Kompleks Rumah Tumbuhan (3° 16′ 14.63″ N, 101° 41′ 11.32″ E) at Universiti Kebangsaan Malaysia, Bangi. Six weeks old *P. minor* plants were selected for this experiment. Selected plants were treated with *Fusarium* along with control (mock infected; sterile distilled water only). Each treatment had two biological replicates. Plant treatments were carried out as explained in our previous report [1].

### 2.2 RNA extraction, quality control and library preparation

Total RNA was isolated using Plant RNA Reagent (Invitrogen, USA) using manufacturer's protocol. Purity and concentration of extracted RNA was measured by Nanodrop spectrophotometer (ND-1000) and Qubit respectively. The integrity of RNA was determined by Bioanalyzer analysis (Agilent 2100) using RNA 6000 chip. The only RNA samples with RNA Integrity Number (RIN) over 7 were selected for further analysis.

Small RNA libraries preparation were carried out using NEBNext® Small RNA Library Preparation kit according to recommended protocols. First, RNA sample was ligated to 3′ SR adaptor and primer hybridization was carried out to prevent dimer formation. Then, 5′ SR adaptor ligation was carried out followed by cDNA synthesis. PCR amplification was carried out to enrich the library of small RNA. Finally, PCR product was subjected to 6% polyacrylamide (PAGE) gel for size selection.

Samples were send to Universiti Malaya, Malaysia for sequencing. Single end reads of 50 base pairs was generated through the Illumina HiSeq 2500™ in Rapid Run mode.

### 2.3 Raw reads pre-analysis and annotation

Further analysis was carried out using CLC Genomics Workbench version 8 (https://www.qiagenbioinformatics.com/). Quality cut-off value used was 20. Minimum percent of bases that had the quality was 90. No ambiguous nucleotide were allowed. Trimming of adapter index sequences were carried out and low quality reads were removed to produce clean reads. Reads with length between 18 to 30 nucleotides were used for annotation, while the rest were discarded (Table 1).

**Table 1:** Pre-analysis of raw reads.

SRA ID	Total number of reads	Total number of reads after trimming	Discard reads
SRX2645684	32,773,784	14,832,557	17,941,227
SRX2645685	53,196,385	36,620,492	16,575,893
SRX2645686	7,410,423	4,375,882	3,034,541
SRX2645687	17,408,948	11,073,982	6,334,966

For annotation of the small RNA, miRNA data set was downloaded from miRBase (version 21) (http://www.mirbase.org/) [2]. The rest of the sequences were mapped against Rfam database (http://www.sanger.ac.uk/science/tools/rfam) [3], [4]. The unannotated sequences were grouped as unknown sequences, since, no match were found in miRBase or Rfam. The annotation results has been summarized in Table 2.

**Table 2:** Raw reads annotation.

SRA ID	Total number of sequence tag	Annotation by miRBase	Annotation by Rfam	Unknown
SRX2645684	2,041,638	4215	123,370	1,913,693
SRX2645685	4,700,646	8048	241,743	4,450,804
SRX2645686	864,735	1796	75,168	707,771
SRX2645687	2,083,536	3424	125,545	1,909,567

## Conflict of interest

All the authors have approved the current manuscript for submission. No conflicts of interest involved.
